# Periodontal pathogen CaZymes: host-pathogen biology, biochemistry and biotechnological exploitation

**DOI:** 10.1080/20002297.2017.1325208

**Published:** 2017-06-20

**Authors:** Graham P. Stafford, Andrew M. Frey, Marianne J. Satur

**Affiliations:** ^a^ Integrated BioSciences, School of Clinical Dentistry, University of Sheffield, Sheffield, UK

## Abstract

One often neglected aspect of the host-pathogen interface is the presence of myriad glycoproteins and the carbohydrate glycans that they present. These are often the first point of contact for bacteria, with the oral cavity being rich in glycoprotein mucins within secretions such as saliva and crevicular fluid. Therefore, unsurprisingly, bacteria have evolved a myriad of enzymes (that one can consider virulence attributes) to access these glycans to allow attachment to host surfaces, which often results in modulation of host-cell behavior but also that bacteria harvest for nutritional purposes.

This talk will summarise our recent work on the role of a range of novel CaZymes from periodontal pathogens in terms of their role in host-bacteria interactions and biofilm formation with potential routes to novel antimicrobials. It will also outline basic biochemical and structural biology work that has identified novel enzymes, lectin domains and biochemical activities that has lead to utilization of these seemingly obscure enzymes in bespoke, specialized biotechnological uses in biopharmaceutical protein analysis that we are currently investigating further.

